# Preoperative conventional chemoradiotherapy versus short-course radiotherapy with delayed surgery for rectal cancer: results of a randomized controlled trial

**DOI:** 10.1186/s12885-016-2959-9

**Published:** 2016-12-01

**Authors:** Tadas Latkauskas, Henrikas Pauzas, Laura Kairevice, Aleksandras Petrauskas, Zilvinas Saladzinskas, Rasa Janciauskiene, Jurate Gudaityte, Paulius Lizdenis, Saulius Svagzdys, Algimantas Tamelis, Dainius Pavalkis

**Affiliations:** 1 Department of Surgery, Lithuanian University of Health Sciences, Kaunas, Lithuania; 2 Department of Oncology and Hematology, Lithuanian University of Health Sciences, Kaunas, Lithuania; 3 Department of Anaesthesiology, Lithuanian University of Health Sciences, Kaunas, Lithuania

**Keywords:** Rectal cancer, Chemoradiotherapy

## Abstract

**Background:**

There still is no evidence which neoadjuvant therapy regimen for stage II–III rectal cancer is superior. The aim of this study was to compare results achieved after long-course chemoradiotherapy (CRT) with short-term radiotherapy (RT) followed by delayed surgery.

**Methods:**

A randomized trial was carried out between 2007–2013. One hundred fifty patients diagnosed with stage II–III rectal cancer were randomized into one of two neoadjuvant treatment arms: conventional chemoradiotherapy (CRT) and short-term radiotherapy (RT) followed by surgery after 6–8 weeks. Primary endpoints of this trial were downstaging and pathological complete response rate. Secondary endpoints were local recurrence rate and overall survival.

**Results:**

The pathological complete response was found in 3 (4.4%) cases after RT and 8 (11.1%) after CRT (*P* = 0.112). Downstaging (stage 0 and I) was observed in 21 (30.9%) cases in RT group vs. 27 (37.5%) cases in CRT group (*P* = 0.409). Median follow-up time was 39.7 (range 4.9–79.7) months. 3-years overall survival (OS) was 78% in RT group vs. 82.4% in CRT group (*P* = 0.145), while disease-free survival (DFS) differed significantly – 59% in RT group vs. 75.1% in CRT group (*P* = 0,022). Hazard ratio of cancer progression for RT patients was 1.93 (95% CI: 1.08–3.43) compared to CRT patients.

**Conclusion:**

Three-years disease-free survival was better in CRT group comparing with RT group with no difference in overall survival.

**Trial registration:**

http://clinicaltrials.gov identifier NCT00597311. January 2008.

## Background

Preoperative conventional chemoradiotherapy (25 × 2 Gy + 5-Fu) with delayed surgery and short-term radiotherapy (5 x5Gy) with immediate surgery are the most common regimens of treatment, causing reduction of local recurrence rate for patients with resectable rectal cancer [1–7]. Two meta-analysis [8, 9] and review [10] showed no differences between these regimens in terms of survival, local recurrence, morbidity, mortality, resectability and the rate of sphincter preservation; however, pathological complete response and toxicity were higher after neoadjuvant chemoradiation.

Radu et al. [11] were the first to present the hypothesis that short-term radiotherapy and delayed surgery could give similar results as conventional CRT. Stockholm III trial reported similar results of complete response, rate of complications and toxicity after preoperative long-course or short-course radiotherapy with delayed surgery [12].

The approach of short-term radiotherapy with delayed surgery has also been evaluated in prospective randomized trial (http://clinicaltrials.gov identifier: NCT00597311) which was carried out between 2007–2013 in the Hospital of Lithuanian University of Health Sciences. Comparison was mainly based on the rates of downstaging after short-term radiotherapy or conventional chemoradiotherapy followed by surgery after 6–8 weeks. Initial results of this trial including the first 83 patients were published previously in 2012 [13]. This is a secondary evaluation of the treatment arms including the same 83 and the total required 150 patients.

## Methods

The study was approved by Kaunas Regional Committee of Ethics of Biomedical Research (Protocol No. 137⁄2006). The inclusion, exclusion criteria and initial results have been reported previously [13]. Completely informed consent was obtained from the patients. Digital examination, endoscopy with biopsy, chest X-ray, abdominal ultrasound and blood analysis were performed. T and N stages were assessed using endorectal ultrasound (ERUS), pelvic computerized tomography (CT) and magnetic resonance imaging (MRI) and patients with resectable stage II or III (T3 N0, T4 N0, Tx N+) rectal cancer were included and blindly randomized into one of the two arms. ERUS, pelvic CT and MRI were repeated before the surgery for re-evaluation of T and N stage and maximal wall thickness. The investigator was not blinded to the pre-treatment ERUS results. The problem of clinical staging was that ERUS was not technically possible for all patients because of localization and size of the tumor and MRI was not made for the first 40 patients.

One hundred fifty patients diagnosed with resectable stage II or III rectal cancer were randomized to two neoadjuvant treatment arms: conventional chemoradiotherapy (CRT; 50 Gy in total administered during a period of 5 weeks, 2 Gy per fraction and two cycles of 5-FU/Leucovarin, 400 mg/m2 of 5-fluorouracil i/v in combination with leucovorin 20 mg/m2 i/v for 1–4 days on the first and on the fifth week) and short-term radiotherapy (RT; 5 fractions of radiotherapy, 5 Gy per fraction, administered each day for 5 days, a dose of 25 Gy in total). Patients were assessed with respect of possible adverse effects of neoadjuvant treatment, along with filling QLQ questionnaires. Data regarding toxicity will be presented in another article.

Both groups underwent surgery in a period of 6 to 8 weeks after a termination of preoperative treatment. Type of surgery was chosen depending on the site of lesion in images of ERUS, pelvic CT and MRI, but final decision was made during the surgery and it was one of the following: abdominoperineal resection, Hartman’s procedure, proctectomy with coloanal anastomosis, or anterior rectal resection. Surgery was performed by the same 6 surgeons specializing in coloproctology. Total mesorectal excision was mandatory during surgery. Adjuvant Chemotherapy of 5-FU (400 mg/m2 i/v and Leucovorin (20 mg/m2 i/v) 1–5 days for 4 cycles every 4 weeks was started within 8 weeks after surgery.

### Follow-up

Patients were examined every 3–6 months during the first 2 years after surgery, and once per year for the following 5 years at least. Evaluation consisted of physical examination, blood tests, abdominal ultrasound, chest radiography and colonoscopy every 6–12 months. CT and MRI were performed in cases of suspected local recurrence or metastasis. No patient was lost during the follow up.

### Statistical analysis

The primary endpoint of this trial was downstaging and pathological complete response rate. In order to calculate the sample size it was assumed that the downstaging rate after chemoradiotherapy would be 40%. To detect a 20% difference between the groups using the *χ*2 test and a significance level of 0.05, the study should include 74 patients (37 patients in each group). Secondary endpoints were local recurrence and overall survival. Secondary endpoints analysis was planned to be performed after recruitement of 150 patients. The *χ*2 test was used for comparison of proportions and the Mann–Whitney *U* test for comparison of continuous variables. Actuarial curves were calculated by the Kaplan–Meier method and were compared by the log rank test. The Cox’s proportional hazards model was used to calculate the hazard ratios and 95% confidence intervals (c.i.) in the univariable analysis. All the tests were two sided. Calculation of time intervals was started from the date of randomization. Patients who had not undergone primary tumour excision or who had distant metastases detected before or during surgery were considered as treatment failures at the time of randomization and were excluded from the study. When calculating the rate of permanent stoma, diverting stoma or stoma after Hartmann’s procedure were excluded, if stoma was reversed later.

## Results

**Fig. 1:**
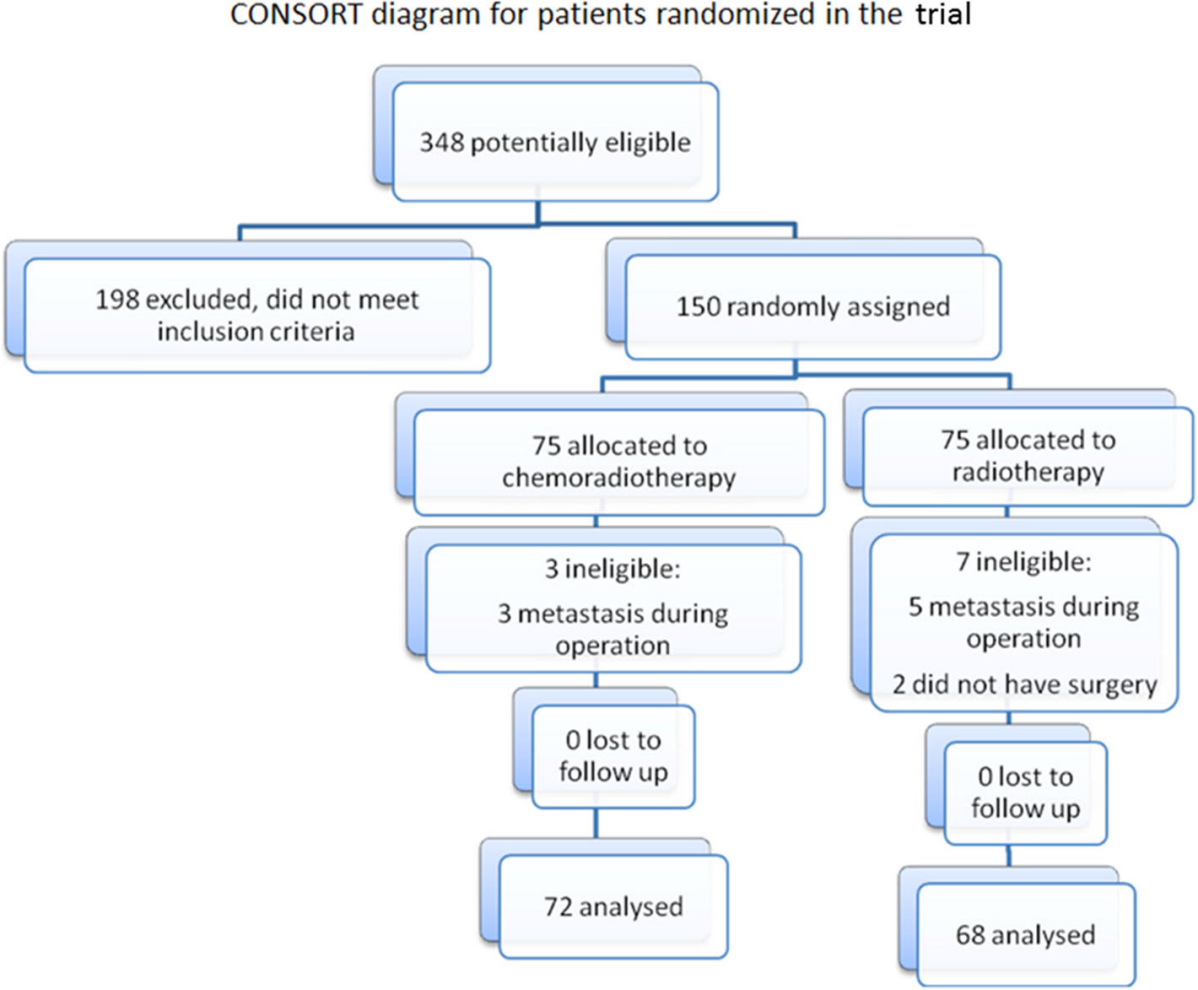
CONSORT diagram for patients randomized in the trial

Amongst 150 randomly assigned patients 10 were excluded from the study due to protocol violation. All eligible patients were included in the statistical analysis (Fig. 1). Patients’ characteristics were similar between the two treatment groups (Table 1).

**Table 1:** Patients main characteristics

Variable	RT *n* = 68	CRT *n* = 72	*P*
Age (years)	65.6 (SD = 9.5)	63.14 (SD = 10.1)	0.141
Gender (%)			
Male	43 (63.2)	50 (69.4)	0.437
Female	25 (36.8)	22 (30.6)	
ASA1 (%)	1 (1.5)	1 (1.4)	
ASA2 (%)	32 (48.5)	31 (43.1)	0.808
ASA3 (%)	33 (50)	40 (55.5)	
Period from the end of			
neoadjuvant therapy till surgery (days)	48.03 (SD = 12.5)	47.14 (SD = 8.6)	0.622
Clinical stage (%)			
II	16 (23.5)	15 (20.8)	0.701
III	52 (76.5)	57 (79.2)	
Tumor distance from anal			
verge (%)			
< 5 cm	34 (50)	30 (41.7)	
5–10 cm	29 (42.6)	37 (51.4)	0.575
11–15 cm	5 (7.4)	5 (6.9)	

### Surgery

RT and CRT groups were comparable regarding the type of surgery and morbidity. Results are presented in Table 2.

**Table 2:** Type of surgery and complications according to treatment groups

Variable	RT (%) *n* = 68	CRT (%) *n* = 72	*P*
Radical surgery	57 (83.8)	64 (88.9)	0.382
Non-radical	11 (16.2)	8 (11.1)	
Postive distal margin (R+)	2	0	
Positive circumferential margin (CRM ≤1 mm)	8	8	
Positive both margins (R and CRM)	1	0	
Postoperative hospital stay (days)	10.06 (SD = 6.9)	9.15 (SD = 3.7)	0.355
Sphincter saving surgery	47 (69.1)	52 (72.2)	0.687
Permanent stoma	27 (39.7)	25 (34.7)	0.542
Defunctioning stoma	41 (60.3)	47 (65.3)	
Anterior resection with anastomosis	35 (51.5)	40 (55.6)	0.959
Proctectomy with coloanal anastomosis	6 (8.8)	7 (9.7)	
Hartman‘s procedure	6 (8.8)	5 (6.9)	
Abdominoperineal resection	21 (30.9)	20 (27.8)	
Complications	24 (35.3)	19 (26.8)	0.277
Anastomotic	4 (5.8)	5 (6.9)	
Wound	7 (10.2)	6 (8.3)	
Stoma	4 (5.8)	1 (1.4)	
Intrasurgical	2 (3.3)	3 (4.3)	
Other	7 (10.2)	4 (5.9)	
Reoperations	1 (1.5)	4 (5.6)	0.235

### Pathology

A histopathological complete response was found in 3 (4.4%) cases after RT and 8 (11.1%) after CRT (*P* = 0.112). Downstaging (stage 0 and I) was observed in 21 (30.9%) cases in RT group vs.27 (37.5%) cases in CRT group (*P* = 0.409). There were more cases with early pathological stage (pT0, pT1) in the CRT group and more cases with pT3 disease in the RT group, however, the difference was not statistically significant (*P* > 0.05). The positive lymph-nodes were found in 25 (36.8%) cases in the RT group and in 18 (25%) cases of CRT group (*P* > 0.05). The majority of patients had a moderately differentiated (G2) tumour (83.6% and 79.7% in the RT and CRT groups, respectively). Groups were comparable in terms of circumferential and distal margin, tumor size, vascular and lymphatic invasion (Table 3).

**Table 3:** Results of postoperative pathological examination

Variable	RT (%) *n* = 68	CRT (%) *n* = 72	*P*
ypT0	3 (4.4)	8 (11.1)	0.112
ypT1	3 (4.4)	5 (6.9)	
ypT2	18 (26.5)	19 (26.4)	
ypT3	44 (64.7)	36 (50)	
ypT4	0	4 (5.6)	
ypN0	43 (64.2)	54 (75)	0.318
ypN1	19 (27.9)	14 (19.4)	
ypN2	6 (8.9)	4 (5.6)	
Differentiation			
G1	6 (9)	5 (7.8)	0.306
G2	56 (83.5)	51 (79.7)	
G3	5 (7.5)	8 (12.5)	
ypL1 (lymphatic)	23 (37.1)	22 (31.9)	0.530
ypV1 (vascular)	19 (31.1)	15 (21.7)	0.233
Distal resection			
margin (mm)	29.83 (SD = 15.1)	31.62 (SD = 17.1)	0.538
Circumferential resection			
margin (mm)	5.02 (SD = 4.44)	7 (SD = 7.19)	0.193
Tumor size (mm)	26.22 (SD = 12.22)	25.13 (SD = 12.48)	0.622

### Long-term oncological results

Median follow-up time was 39.7 (range 4.9–79.7) months. During the time of observation 31 patients had died: 17 (25%) in RT group and 14 (19.4%) in CRT group (*P* > 0.05). Hazard ratio of death for RT patients compared to patients in CRT group was 1.64 (95% CI: 0.8–3.43). Cancer progression was observed in 16 (25%) cases in RT group vs. 13 (18.3%) cases in CRT group (*P* > 0.05). The rate of local recurrence between the groups was: 2 (3.1%) cases in RT group vs. 4 (5.6%) in CRT group (*P* > 0.05). During the follow-up distant metastases developed in 14 (21.9%) cases after RT and in 9 (12.7%) cases after CRT (*P* > 0.05). Hazard ratio of distant metastases for RT patients compared to CRT patients was 2.2 (95% CI: 0.95–5.10). Causes of death and cancer progression are summarized in Table 4.

**Table 4:** Causes of death and cancer progression

Variable	RT (%) *n* = 68	CRT (%) *n* = 72	*P*
Death	17 (25)	14 (19.4)	0.429
related to cancer	12	12	
not related to cancer	3	0	
unknown	2	2	
Progression of rectal cancer	16 (25)	13 (18.3)	0.345
local recurrence alone	0	3 (4.2)	
distant metastases alone	12 (18.8)	8 (11.3)	
local recurrence with distant metastases			
in liver and lungs	2 (3.1)	1 (1.4)	
metastatic tumor in peritoneal cavity	2 (3.1)	1 (1.4)	

**Fig. 2:**
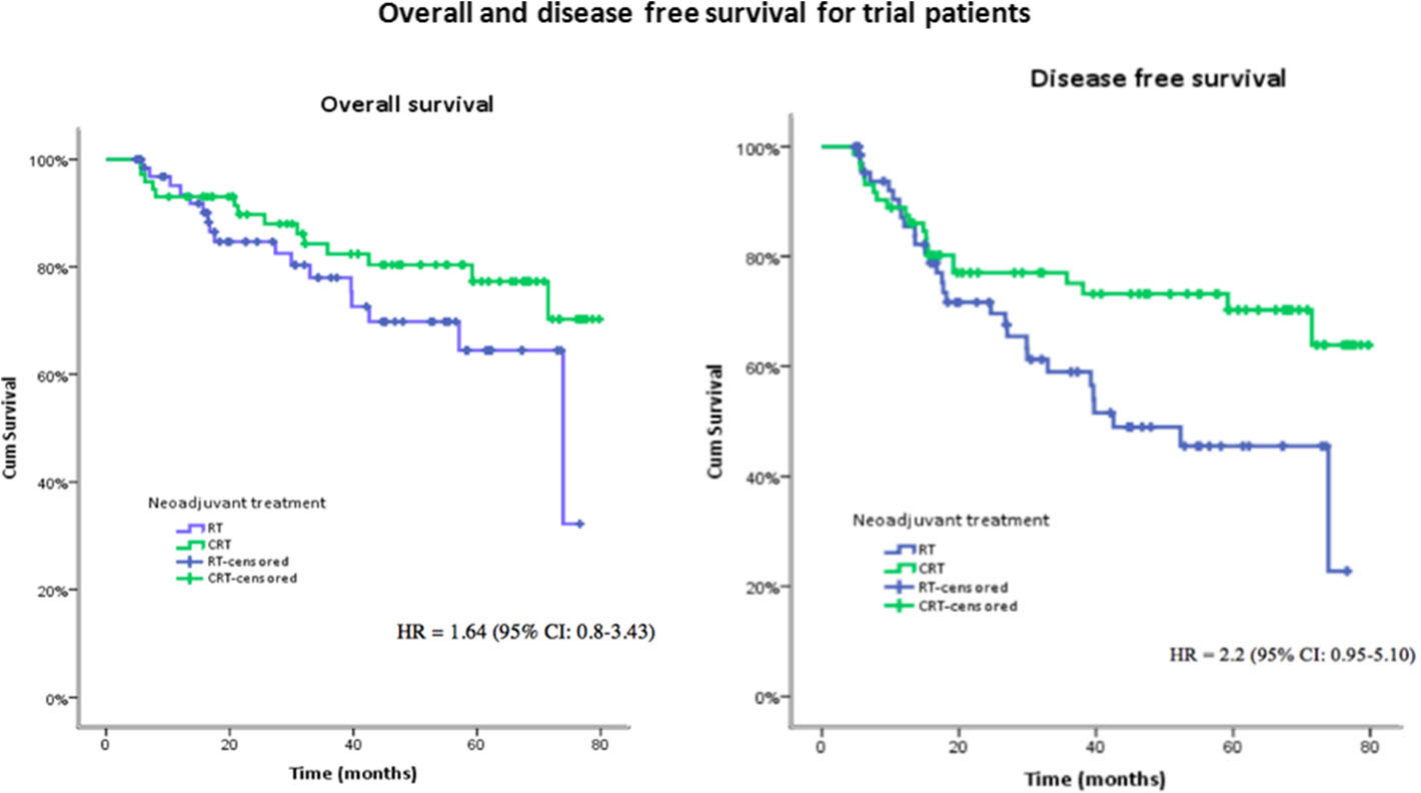
Overall and disease free survival rates for the trial patients according to treatment received

Three- years OS was 78% in RT group vs. 82.4% in CRT group (*P* = 0,145), while DFS was 59% in RT group vs. 75.1% in CRT group (*P* = 0,022) (Fig. 2). Hazard ratio of cancer progression (distant and local) for RT patients compared to CRT patients was 1.93 (95% CI: 1.08–3.43).

## Discussion

Currently there is no optimal neoadjuvant treatment regimen for locally advanced rectal cancer yet. According to ESMO clinical practice guidelines of 2013, conventionally fractionated chemoradiotherapy (25 × 2 Gy) with delayed surgery or short-course radiotherapy (5 ×5 Gy) with surgery in 1 week for resectable rectal cancer are recommended [14]. These recommendations are based on the results of well known trials [1], but the problem persists that these two treatment options are quite different, resulting in different treatment policies among countries or even specialists in the same country.

Two meta-analyses revealed no differences between these regimens in terms of the rates of survival, local recurrence, morbidity, mortality, resectability and the rate of sphincter preservation, and only pathological complete response and toxicity were higher after neoadjuvant chemotherapy [8, 9]. Bujko et al. compared neoadjuvant short-course radiotherapy (RT) followed by surgery within 7 days with conventional long-course chemoradiotherapy (CRT) and found that 4 years overall, disease-free survival rates and local recurrence rate did not differ significantly between the groups [6]. Complete response rate was higher in CRT group: 16.1% *vs* 0.7%, tumor involvement of the circumferential margin was 4% after chemoradiotherapy *vs* 13% after short-course radiotherapy (*P* < 0.05), but the toxicity (incidense of III-IV grade adverse effects) rate was also significantly higher 18.2% *vs* 3.2% in CRT vs RT group, respectively.

Retrospective data from Radu et al. showed that pathological complete response and local disease control have been similar between short-term radiotherapy with delayed surgery (6–8 weeks) and long-course chemoradiotherapy, with low rates of toxicity in both groups [11]. Stockholm III trial compared short-course radiotherapy (5 × 5 Gy) with long-course radiotherapy (25 × 2 Gy) without chemotherapy both with delayed surgery and reported 12.5% complete response rate after short-course RT compared with 5% after long-course RT (*P* < 0.05) [12].

On the contrary, we found that complete response rate was 4.4% after RT vs. 11.1% after CRT (*P* > 0.05) and downstaging (pathological stage 0 and I) was observed in 30.9% cases in RT group vs. 37.5% cases in CRT group. According to Stockholm III trial results, pathological stage 0 and I was found in 45% cases after short-course and in 30% cases after long-course RT. These differences could be explained as follows: chemotherapy was added to long-course radiotherapy according the protocol of our trial.

Disease progression was observed in 25% of cases in RT group vs. 18.3% of cases in CRT group. The rate of local recurrence between groups was: 3.1% of cases in RT group *vs* 5.6% of cases in CRT group, respectively. 3 year overall survival was comparable between the groups: 82.4% after CRT vs. 78% after RT, but disease-free survival was significantly better after CRT (75.1%) than after RT (59%).

Distant metastases (undetected preoperatively) were found intraoperatively for 5 patients (6.7%) after short-course radiotherapy and 3 (4%) patients after chemoradiotherapy. These patients were excluded from the analysis of long-term results. The question if this distant spread was missed during primary investigation is open, because abdominal ultrasound (not CT) as a routine method of investigation was used according our trial protocol, but it could also be the result of early cancer progression, and in that case DFS would be slightly worse.

Local recurrence rate for resectable rectal cancer after surgery with TME is quite low. The TME approach increases the likelihood of clear circumferential resection margins which corresponds to decreased rates of pelvic recurrence [15]. The majority of rectal cancer deaths is likely to be associated with distant metastases, not from local recurrence. This could explain why no survival benefit was found in the majority of trials comparing various regimens of neoadjuvant treatment for rectal cancer [2]. Chemotherapy controlling distant progression of the disease could be beneficial for these patients, but FFCD study did not prove any advantage for the addition of 5-Fu to RT in terms of DFS or OS [7].

There is no discussion that low-risk rectal cancer patients for whom imaging allows safe R0 resection should go for initial surgical treatment, while preoperative chemoradiotherapy should be administered for patients with high risk of local recurrence (threatened resection margins), [16]. A major concern is a large group of patients with intermediate-risk rectal cancer (T3, >3 mm CRM), for whom neoadjuvant chemoradiotherapy appears to be potential overtreatment, but preoperative systemic chemotherapy controlling distant spread could be beneficial. A pilot trial from Memorial Sloan Kettering Cancer Center reported promising results of selective use of chemoradiation for patients with intermediate-risk rectal cancer. Results from this pilot study served as a background to initiate the currently undergoing PROSPECT trial [16].

Despite discussions most authors agree that careful staging and individualized treatment approach including selective combination of surgery, chemo- or radiotherapy, should be recommended for patients with rectal cancer.

## Conclusion

According to the results of the randomized controlled trial, 3-years DFS was better in CRT group compared with RT group with no difference in OS. Surgical recovery and perioperative morbidity were similar between the groups.

## Abbreviations

CRT: Long-course chemoradiotherapy
CT: Computerized tomography
DFS: Disease-free survival
ERUS: Endorectal ultrasound
MRI: Magnetic resonance imaging
OS: Overall survival
RT: Short-term radiotherapy

## References

[1] Preoperative versus postoperative chemoradiotherapy for locally advanced rectal cancer: results of the German CAO/ARO/AIO-94 randomized phase III trial after a median follow-up of 11 years. J Clin Oncol. 2012;30(16):1926-33. 10.1200/JCO.2011.40.1836 22529255

[2] Peeters KC, Marijnen CA, Nagtegaal ID, Kranenbarg EK, Putter H, Wiggers T (2007). The TME trial after a median follow-up of 6 years: increased local control but no survival benefit in irradiated patients with resectable rectal carcinoma. Ann Surg.

[3] Cedermark B, Johansson H, Rutqvist LE, Wilking N (1995). The Stockholm I trial of preoperative short term radiotherapy in operable rectal carcinoma. A prospective randomized trial. Stockholm Colorectal Cancer Study Group. Cancer.

[4] Martling A, Holm T, Johansson H, Rutqvist LE, Cedermark B (2001). The Stockholm II trial on preoperative radiotherapy in rectal carcinoma: long-term follow-up of a population-based study. Cancer.

[5] Bosset JF, Collette L, Calais G, Mineur L, Maingon P, Radosevic-Jelic L (2006). Chemotherapy with preoperative radiotherapy in rectal cancer. N Engl J Med.

[6] Bujko K, Nowacki MP, Nasierowska-Guttmejer A, Michalski W, Bebenek M, Kryj M (2006). Long-term results of a randomized trial comparing preoperative short-course radiotherapy with preoperative conventionally fractionated chemoradiation for rectal cancer. Br J Surg.

[7] Gerard JP, Conroy T, Bonnetain F, Bouche O, Chapet O, Closon-Dejardin MT (2006). Preoperative radiotherapy with or without concurrent fluorouracil and leucovorin in T3-4 rectal cancers: results of FFCD 9203. J Clin Oncol.

[8] Latkauskas T, Paskauskas S, Dambrauskas Z, Gudaityte J, Saladzinskas S, Tamelis A (2010). Preoperative chemoradiation vs radiation alone for stage II and III resectable rectal cancer: a meta-analysis. Colorectal Dis.

[9] Ceelen W, Fierens K, Van Nieuwenhove Y, Pattyn P (2009). Preoperative chemoradiation versus radiation alone for stage II and III resectable rectal cancer: a systematic review and meta-analysis. Int J Cancer.

[10] De Caluwe L, Van Nieuwenhove Y, Ceelen WP (2013). Preoperative chemoradiation versus radiation alone for stage II and III resectable rectal cancer. Cochrane Database Syst Rev.

[11] Radu C, Berglund A, Pahlman L, Glimelius B (2008). Short-course preoperative radiotherapy with delayed surgery in rectal cancer - a retrospective study. Radiother Oncol.

[12] Pettersson D, Cedermark B, Holm T, Radu C, Pahlman L, Glimelius B (2010). Interim analysis of the Stockholm III trial of preoperative radiotherapy regimens for rectal cancer. Br J Surg.

[13] Latkauskas T, Pauzas H, Gineikiene I, Janciauskiene R, Juozaityte E, Saladzinskas Z (2012). Initial results of a randomized controlled trial comparing clinical and pathological downstaging of rectal cancer after preoperative short-course radiotherapy or long-term chemoradiotherapy, both with delayed surgery. Colorectal Dis.

[14] Glimelius B, Tiret E, Cervantes A, Arnold D (2013). Rectal cancer: ESMO Clinical Practice Guidelines for diagnosis, treatment and follow-up. Ann Oncol.

[15] Heald RJ, Ryall RD (1986). Recurrence and survival after total mesorectal excision for rectal cancer. Lancet.

[16] Schrag D, Weiser MR, Goodman KA, Gonen M, Hollywood E, Cercek A, et al. Neoadjuvant Chemotherapy Without Routine Use of Radiation Therapy for Patients With Locally Advanced Rectal Cancer: A Pilot Trial. J Clin Oncol. 2014;13. 10.1200/JCO.2013.51.7904 PMC5795691 24419115

